# Commentary: P2X7 receptor modulation is a viable therapeutic target for neurogenic pain with concurrent sleep disorders

**DOI:** 10.3389/fnins.2023.1293174

**Published:** 2023-11-30

**Authors:** Sareena Shah, Karishma Kondapalli, Nabeel Rasheed, Xiang-Ping Chu

**Affiliations:** Department of Biomedical Sciences, University of Missouri–Kansas City School of Medicine, Kansas, MO, United States

**Keywords:** P2X7R, neurogenic pain, sleep, chronic pain, ligand-gated ion channel

Chronic neuropathic pain can severely impact people's quality of life, influencing their mood, activities of daily living, sleep, and cognition. In comparison to the general population, patients with chronic pain have a higher risk of experiencing major depressive disorder (MDD) and sleep disturbances; in turn, MDD and impaired sleep can contribute to the worsening of chronic pain (Emery et al., 2013). One meta-analysis found that employing non-pharmacologic interventions for chronic low back pain resulted in improved sleep outcomes (Craige et al., 2023). In this regard, improving sleep quality, with or without the use of medication, is an important factor in addressing chronic pain. The central nervous system (CNS) hosts many receptor types that mediate various signals; the P2X family of receptors plays a particularly significant role in neuromodulation. P2X7 receptors (P2X7R) are purinergic ligand-gated ion channels, meaning their activation depends on binding from ATP or ADP. These receptors are important for physiological maintenance such as memory, sleep, and cognition, but can also play a part in cellular injury and inflammation (Burnstock, 2015).

Specific subtypes of P2X receptors are involved in the neurotransmission of pain: while P2X3 and P2X4 are respectively located in sensory neurons and microglia, P2X7 possesses a larger distribution in the CNS due to its presence in both sensory neurons and microglia (Inoue and Tsuda, 2020). These receptors are important components of pain, immune, and inflammatory responses within the body (Jacobson et al., 2023). The pro-inflammatory functions of P2X7R contribute significantly to chronic pain (Ren and Illes, 2022). Along with sleep disturbances in neuropathic pain, P2X7 receptors have been found to be present in psychiatric conditions, such as MDD, bipolar disorder, and schizophrenia, and neurodegenerative disorders, such as Alzheimer's, Parkinson's disease, amyotrophic lateral sclerosis, and multiple sclerosis (Zhang et al., 2022).

In terms of therapeutic benefit, recent studies turn their focus toward P2X7R antagonists, which vary in their design based on ligand, structure, or fragment (Zhang et al., 2023). This is because the levels of P2X7R are upregulated in pathophysiological conditions (Lee and Kim, 2022). A recent study has shown that glial cell P2X7R expression in the hippocampus was increased after inducing a chronic stress response in mice models, and P2X7R inhibition was consequently associated with a decrease in mice stress behaviors (Yue et al., 2017). This association can play role in influencing the quality and quantity of sleep in patients with chronic pain, which may be worsened in the context of chronic stress. Another study revealed that rats with diabetic neuropathy had increased expression of the P2X7R and inhibiting the P2X7R in these animals decreased displays of pain (Chen et al., 2022). Furthermore, mice models with disruption of P2X7R genes demonstrated significantly less inflammatory and neuropathic hypersensitivity to various pain stimuli compared to their wild-type counterparts (Chessell et al., 2005). In fact, one study used a chronic-constriction injury model in mice to investigate the role of the P2X7R in mediating sleep disturbances exacerbated by neuropathic pain (Li et al., 2023). Ultimately, the P2X7R serves as a gateway for novel advances in pharmacologic therapy to address sleep disturbances associated with neuropathic pain (Li et al., 2023). Targeting this receptor can improve the quality of life for patients with neuropathic pain, as well as set the stage for researching similar therapies for other chronic In review pain conditions, including malignancy, neurodegenerative disease, and psychiatric illnesses. Therefore, it is necessary to further investigate the relationships between the P2X7R and pain signaling.

Our study discusses findings from a recent study by Li et al. (2023), which was published in Frontiers in Neuroscience and discusses the use of various wet lab techniques including electroencephalogram (EEG) recordings, electromyography (EMG) recordings, local field potential (LFP) recordings, immunofluorescence, sample extraction, and [1H-13C]-NMR spectroscopy to investigate the effect of neuropathic pain (NP) on neuronal activity during sleep. The mice were divided into four groups: Sham, Sham + A-740003 (a selective P2X7R antagonist), chronic-constriction injury (CCI) group, and CCI + A-740003. Mice were given A-740003 or DMSO (which served as control injection) daily for 3 days prior to the measurement. The P27XR inhibitor was a selective antagonist with the following cellular makeup: A-740003 [N-(1-{[(Cyanoimino) (5-quinolinylamino)methyl] amino} −2,2-dimethylpropyl)-2-(3,4-dimethoxyphenyl) acetamide] (*i.p*.; 0.5 mL, 180 mg/kg, dissolved in 1% DMSO, 44% PEG-300, 5.5% Tween-80, and 49.5% saline) (A0862, Sigma-Aldrich, Germany). The PEG-300 was used due to its ability to improve the solubility of A-740003 and compatibility with organic components.

Pain related behaviors were evaluated using the mechanical paw withdrawal threshold (MWT) and the paw thermal withdrawal latency (PWL) to examine the threshold of pain mice on certain days before and after surgery. These tests were conducted on the first day before surgery and then again on the 3rd, 5th, 7th, 14th, and 24th day after surgery. Mechanical hyperalgesia and heat allodynia were significantly decreased from postoperative day 3 until 3 weeks after surgery in the CCI group when compared to the sham group. The A-740003 treatment attenuated the CCI-induced mechanical hyperalgesia and heat allodynia on the days 7th, 14th, and 21st post-surgery. The difference between mechanical and heat perception when comparing the sham group and mice given A-740003 alone was not statistically significant. Collectively, the data suggests that alleviation of CCI induced pain hyperalgesia can be achieved through inhibition of P2XR7.

Twenty-four-hour recordings of EEG and EMG were used to confirm the impact of the P2X7R on sleep/awake patterns after A-74003 administration. Mice in the CCI group were compared to the sham group and results demonstrated that the CCl group spent less time in NREM sleep and more time in wakefulness during both light and dark phases; however, time spent in REM sleep was not different between the two. The administration of A-740003 resulted in a notable reduction in the decrease of NREM sleep time and an increase in wake time in chronic pain mice, particularly during the light phase. Additionally, there was no significant disparity detected in the sleep/wakefulness states between the group treated with A-740003 and the control group. Ultimately, it was determined that NREM sleep after chronic constriction injury was promoted by the inhibition of P2X7R.

Further, the study concluded that inhibition of P2X7R decreased P2X7R-activated microglia in the cortex after chronic-constriction injury using immunohistochemical methods. This test was performed on the 14th day after CCI and demonstrated that microglia expressed by P2X7R+-Iba1+ in CCI mice was significantly higher than in the sham mice. Moreover, A74003 administration resulted in a decrease in the number of P2X7R+ –Iba1+ microglia of the CCI group.

LFP power spectra were recorded in the ventral posterior nucleus (VP) and primary somatosensory cortex (S1). After CCI intervention, relative power percentage of delta oscillation decreased in VP and S1 in comparison to the sham group. Delta oscillations improved when treated with in review A-740003. Mice with CCI had a higher percentage for the alpha oscillation and theta oscillations in the VP, when compared to the control group. Adding A-740003 reversed the alpha oscillations but not the theta oscillations. There was an increase in the relative power percentage of beta oscillations after CCI intervention. A-740003 intervention reversed this effect in S1 but not in VP. The results suggested that restoration of faster oscillations could be promoted by P2X7R inhibition.

Additionally, the study used coherences of LFP (1–50 Hz) in the VP and S1 to conclude that inhibition of P2X7R decreased the coherence of local field potential in ventral posterior nucleus and primary somatosensory cortex after chronic-constriction injury. When compared to the sham group, there was a significant increase in total coherence in the CCI group, which was reversed by adding the P2X7R inhibitor A-740003. The results of the study suggest that the sleep disturbance induced by NP is probably linked to alterations in coherence between VP and S1, and this effect can be reversed by P2X7R.

Levels of Glu4, Gln4, Glu3, and Glx3 were monitored in the thalamus (TH) and parietal cortex (PC) using NMR to evaluate glucose levels after CCI model. Compared to the control group, the metabolism of CCI group showed increased 13C enrichments for Glu4, Gln4, Glu3 and Glx3 in both regions. However, after A740003 administration, only some changes were recovered, including Glu4, Gln4, Glu3, and Glx3 in the TH and Glu4, Gln4, and Glx3 in the PC. Overall, most metabolites can be restored after CCI with the inhibition of P2X7R, which is assumed to correlate with CCI-induced sleep disorder. Patients with chronic pain who experience sleep disturbances have been shown to experience a greater severity of pain, longer durations of pain, greater disability, and lower physical activity levels than counterparts without sleep disturbances (Husak and Bair, 2020). In addition, these patients are at greater risk for concurrent depression, anxiety, and suicidal ideation. Often times, repeated sleep disturbances can also contribute to built-up fatigue, impairing cognitive function and reducing quality of life (Irwin, 2015). There is a clear need for research investigating therapeutic options for managing concomitant chronic pain and sleep disorders.

It is important to note that the present study did not compare the proposed P2X7R inhibition with a positive control, which should be an avenue for future investigation. Additionally, there is not a discussion of potential harms of purine antagonism included in the text. Previous studies have reported potential detrimental effects of P2X7R inhibition in the pathogenesis of autoimmune disorders (Faliti et al., 2019; Mellouk et al., 2022). Additionally, there may be concern for poor wound healing, effects on neuromodulators, and loss of immune protection as other side effects (Jin et al., 2014; Ribeiro et al., 2019; Grassi and De Ponte Conti, 2021). Further research is needed to investigate potential side effects and toxicities of P2X7R inhibition as a therapeutic tool.

Neuropeptides have established their role in sleep regulation while also being associated with chronic pain conditions (Saper et al., 2005; Furlan et al., 2016). As discussed in the study published by Li et al. (2023), P2X7R inhibition is a therapeutic avenue to increase NREM sleep and alleviate pain and hyperalgesia. The P2X7 receptor is a fascinating target due to its wide expression in microglia, which have recently been shown to serve a critical role in neuronal activity during the pain and sleep cycle (Xiao et al., 2022). P2X receptors are a main target for ATP extracellular signaling pathways, which plays a critical role in nociception and chronic pain mechanisms (Moldofsky et al., 2011). P2X7R specifically is located in peripheral and central immune cells and initiates immune system activation through macrophages, cytokine production, reactive oxygen species, and excitatory glutamate release, which are all contributors of chronic pain processes (Bernier et al., 2018). Overall, P2X7R antagonists are a well-studied class of therapeutics due to its primary role in pain mechanisms.

Along with P2X7R inhibition, early studies have also investigated other targets for pharmaceutical interventions. One therapeutic which has been studied for treating chronic pain and sleep disturbances is In review pregabalin, which is an anticonvulsant medication commonly used to treat neuropathic pain associated with diabetic neuropathy, spinal cord injury, postherpetic neuralgia, and fibromyalgia (Deurveilher et al., 2021). Pregabalin is a gamma-amino-butyric acid (GABA) analog which mimics GABAergic inhibition by binding to the alpha2 delta subunit on neuronal voltage-gated calcium channels in the CNS, subsequently reducing membrane fusion and exocytosis of neurotransmitters (Cross et al., 2022). Recently, a systematic review by Husak and Bair (2020) found that pregabalin was the most commonly studied pharmaceutical option for concomitant chronic pain and sleep disturbances, being reported in 5 studies evaluating patients with fibromyalgia. In all 5 studies, patients taking pregabalin showed significant improvements in both pain and sleep when compared with placebo. Pregabalin has also been shown to be useful in improving sleep quality for patients with generalized anxiety disorder, neuralgia, and epilepsy (Micó and Prieto, 2012). An additional study by Moldofsky et al. (2011) gave a low dose of cyclobenzaprine to patients to improve sleep physiology in patients with fibromyalgia experiencing poor sleep patterns. This double-blind, placebo-controlled trial included 37 patients with fibromyalgia and prescribed an increasing dose of cyclobenzaprine ranging from 1–4 mg over the course of 8 weeks. Results suggested that cyclobenzaprine improved fibromyalgia symptoms at night resulting in better sleep for these patients. Though fibromyalgia is a specific condition, it would be interesting to explore if administration of cyclobenzaprine would improve symptoms in other conditions causing neuropathic pain.

Along with P2X7R, other P2X receptors may be involved with chronic pain and sleep disorders (Bernier et al., 2018; Ren and Illes, 2022). P2X4R on the dorsal root ganglion and spinal cord are stimulated by chronic nerve injury and inflammation, rather than by acute pain signals (Aby et al., 2018). The activation of P2X3 and P2X2/3 receptors in the nerve fibers of rodent tooth pulp demonstrated pain response behaviors and brainstem activation (Adachi et al., 2010). In one study, antagonizing P2X3R in the carotid body stabilized abnormal breathing patterns and reduced inflammation in rats with heart failure; this therapy may introduce a novel method of improving heart failure-associated orthopnea and poor sleep quality (Lataro et al., 2023). P2X7R has important potential as a target for pain and sleep disorders, but investments in further studies regarding the other P2X receptor subtypes' therapeutic implications are warranted.

Ultimately, this study offers valuable insights into the regulatory role of purine receptor modulation and its receptor in sleep and its relationship with chronic pain conditions. These findings have the potential to open new avenues for the development of innovative therapeutic interventions for managing sleep disturbances in individuals with chronic pain. In light of these promising results, future research in this area is warranted to further explore the therapeutic potential of purine receptor signaling pathways (or other relevant pathways) in addressing the complex interplay between sleep and pain regulation.

## Author contributions

SS: Conceptualization, Data curation, Formal analysis, Writing—original draft. KK: Conceptualization, Data curation, Formal analysis, Writing—original draft. NR: Formal analysis, Writing—original draft. X-PC: Conceptualization, Supervision, Validation, Writing—review & editing.
