# Supplementary material for: Commentary: P2X7 receptor modulation is a viable therapeutic target for neurogenic pain with concurrent sleep disorders
Source: Front Neurosci. 2023 Nov 30;17:1293174. doi: 10.3389/fnins.2023.1293174 (PMC10720246; doi:10.3389/fnins.2023.1293174)
Supplement: Supplementary file 1 [file Data_Sheet_1.PDF]

Commentary: P2X7 receptor modulation is a viable therapeutic target for neurogenic pain with concurrent sleep disorders

Introduction

- Chronic neuropathic pain can severely impact people’s quality of life
- The P2X family of receptors plays a particularly significant role in neuromodulation. P2X7 receptors are purinergic ligand-gated ion channels, meaning their activation depends on binding from ATP or ADP.
- These receptors are important for physiological maintenance such as memory, sleep, and cognition, but can also play a part in cellular injury and inflammation

Findings

- A recent study by Li et al. published in Frontiers in Neuroscience discusses the use of various wet lab techniques to investigate the effect of neuropathic pain (NP) on neuronal activity during sleep
- Fndings from this study suggest that neuropathic pain can effect neuronal activity linked to sleep.
- This conclusion allows for inquiry of a new target for the development of sleep disturbances under chronic pain conditions.

Perspective

- P2X7R antagonists are a well-studied class of therapeutics due to its primary role in pain mechanisms.
- Future studies should investigate pregablin and cyclobenzaprine for treatment of NP
- Other P2X receptor subtypes’ should also be included in future studies
- Future research in this area is warranted to further explore the therapeutic potential of purine receptor signaling pathways (or other relevant pathways) in addressing the complex interplay between sleep and pain regulation.
